# The effect of COVID-19 on trauma system in one city of China

**DOI:** 10.1186/s13049-020-00752-7

**Published:** 2020-06-23

**Authors:** Fenglu Yang, Xiao Lu

**Affiliations:** 1 Department of Emergency Medicine, Second Affiliated Hospital, Zhejiang University School of Medicine, No.88, Jiefang Road, Hangzhou, 310009 China; 2 Pre-hospital care center of Hangzhou, Hangzhou, China

## Abstract

The aim of this letter to the editor was to report the effect of COVID-19 on the trauma system in one large city in China. We aimed to elucidate what happened to the prehospital system of the city and level 1 trauma centers during the epidemic, especially after the city lockdown. As the data showed, the lockdown reduced the volume of trauma patients throughout the trauma system, and prehospital system of the city during the epidemic was also obviously changed.

The city of Hangzhou is a metropolis with over 12 million people. Since the onset of the COVID-19 pandemic in China at the end of December 2019, the municipal government implemented strict measures to control the epidemic, including locking down the whole city on January 26, 2020. Although there have been only 181 confirmed cases in the city to date, COVID-19 has had pronounced effects on the trauma system.

As shown in Fig. 1, the number of major trauma patients who were transferred from prehospital care to the trauma centers decreased from 1312 in 2019 to 796 in 2020, a 39.2% reduction after the lockdown. The number of cases doubled after the lockdown was eased at the beginning of March. The number of cases returned to normal in April, during which it exceeded the number of trauma patients in April 2019. As shown in Fig 2, there was a significant reduction found in the number of male trauma patients after the lockdown. The variation in the number of patients admitted to one trauma intensive care unit of the largest level 1 trauma center was as follows: the number of severe trauma patients (ISS > 16) who needed critical care decreased from 42 to 6 in a month after the lockdown.

**Fig. 1:**
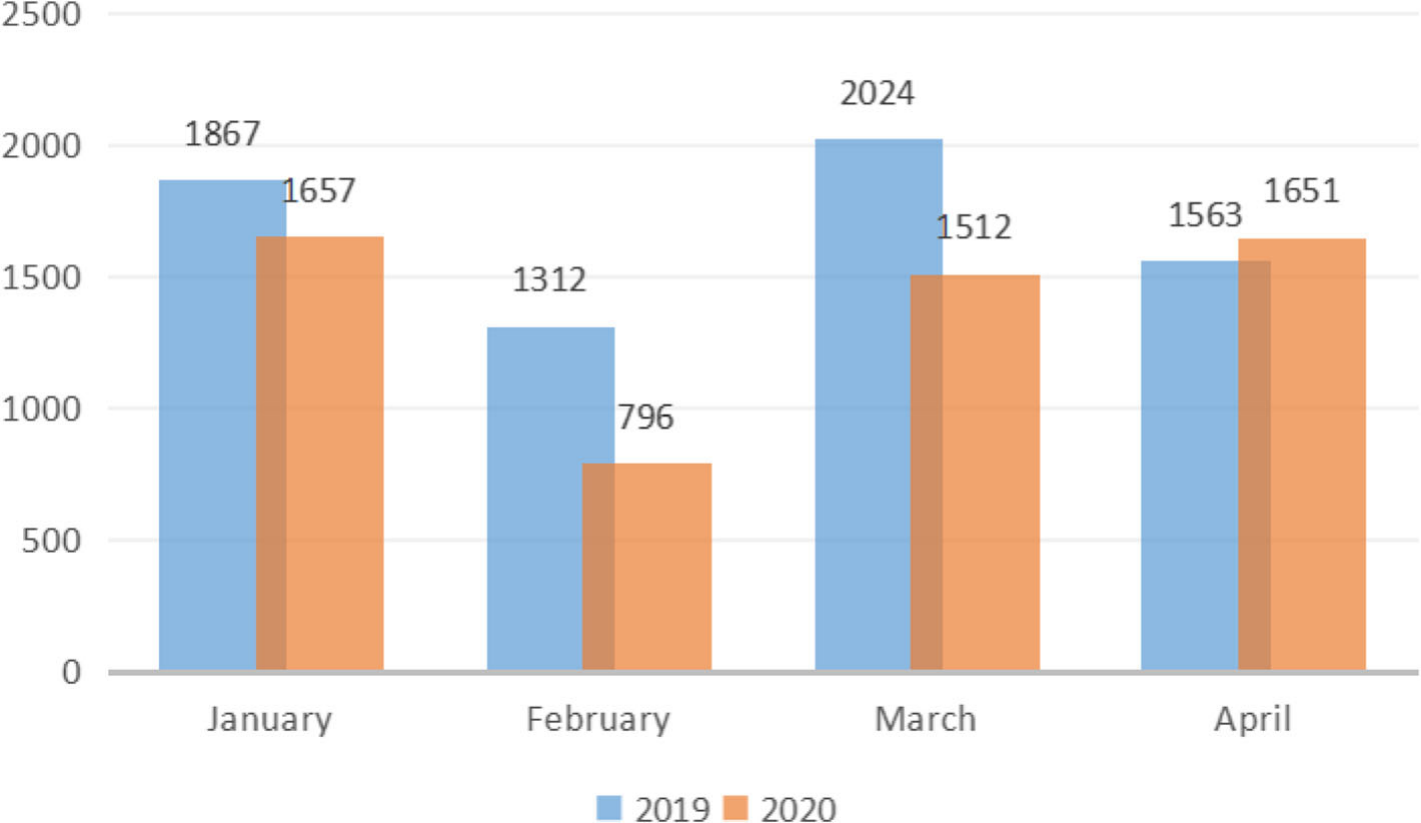
Variation in the number of major trauma patients needing prehospital care and transferred after the lockdown

**Fig. 2:**
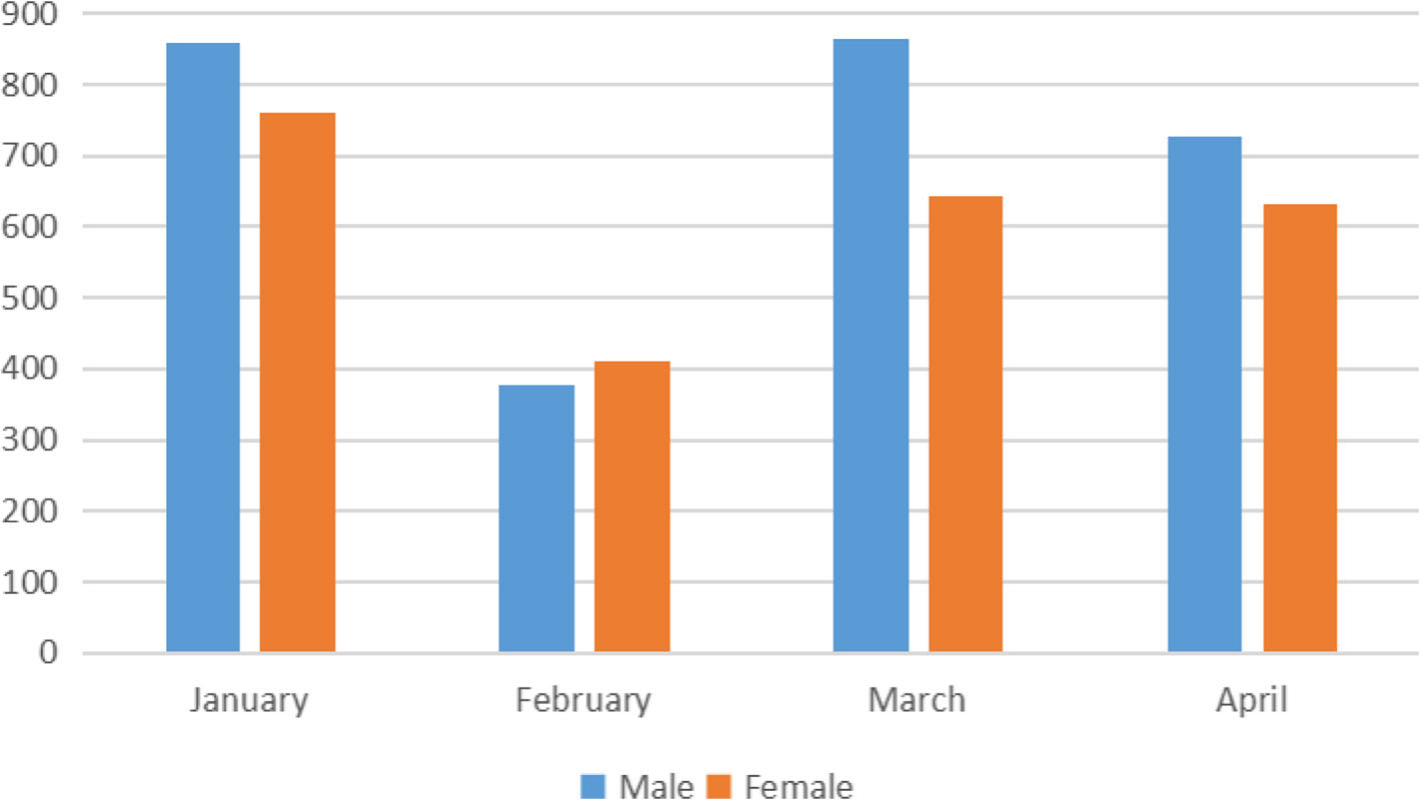
Variation in the sex ratio of the trauma patients needing prehospital care and transferred after the lockdown

COVID-19 quarantines have reduced the volume of trauma patients throughout the system. Despite the significant reduction in admissions during level-3 lockdown, hospitals should continue to provide full services until resource limitations become unavoidable. During this pandemic, essential medical and surgical services must be carried out while minimizing the risk of disease transmission to health care workers. The greatest reductions were seen in major injuries and males: this suggests that males are at high risk of non-lockdown activities, such as road traffic crashes, work, school, and sports [1]. Medical trauma has continued to occur in all of the age groups at all levels of severity, albeit at greatly reduced volumes. In terms of resource allocation, hospitals should continue to provide full services until significant resource restriction occurs [2].The prehospital system of the city during the epidemic also changed markedly. In essence, emergency medical services capacity decreased as the many confirmed and suspected cases need to be transferred to appointed hospitals in negative pressure ambulances. There are five level-1 trauma centers in this city. Severe trauma patients could receive a high quality of treatment, but the waiting time for emergency surgery increased considerably because patients are required to undergo an oropharyngeal swab and, when appropriate, a chest CT scan in order to test them for COVID-19.

## Data Availability

Not applicable.

## Abbreviations

ISS: Injury Severe Score
COVID-19: Corona Virus Disease 2019
